# Strengthening Antimicrobial Resistance Diagnostic Capacity in Rural Rwanda: A Feasibility Assessment

**DOI:** 10.5334/aogh.3416

**Published:** 2021-08-06

**Authors:** Grace Umutesi, Lotta Velin, Moses Muwanguzi, Kara Faktor, Carol Mugabo, Gilbert Rukundo, Aniceth Rucogoza, Marthe Yankurije, Christian Mazimpaka, Jean de Dieu Gatete, Cyprien Shyirambere, Bethany Hedt-Gauthier, Robert Riviello, Tharcisse Mpunga, Emil Ivan Mwikarago, Fredrick Kateera

**Affiliations:** 1 Partners In Health/Inshuti Mu Buzima, Rwinkwavu, Rwanda; 2 Department of Global Health, University of Washington, Seattle, WA, USA; 3 Program in Global Surgery and Social Change, Harvard Medical School, Boston, MA, USA; 4 Surgery and Public Health, Department of Clinical Sciences Lund, Faculty of Medicine, Lund University, Sweden; 5 National Reference Laboratory, Rwanda Biomedical Centre, Ministry of Health, Kigali, Rwanda; 6 Department of Global Health and Social Medicine, Harvard Medical School, Boston, MA, USA; 7 Center for Surgery and Public Health, Brigham and Women’s Hospital, Boston, MA, USA; 8 Ministry of Health, Kigali, Rwanda

## Abstract

**Introduction:**

Antimicrobial resistance (AMR) is a global public health threat. Worse still, there is a paucity of data from low- and middle-income countries to inform rational antibiotic use.

**Objective:**

Assess the feasibility of setting up microbiology capacity for AMR testing and estimate the cost of setting up microbiology testing capacity at rural district hospitals in Rwanda.

**Methods:**

Laboratory needs assessments were conducted, and based on identified equipment gaps, appropriate requisitions were processed. Laboratory technicians were trained on microbiology testing processes and open wound samples were collected and cultured at the district hospital (DH) laboratories before being transported to the National Reference Laboratory (NRL) for bacterial identification and antibiotic susceptibility testing. Quality control (QC) assessments were performed at the DHs and NRL. We then estimated the cost of three scenarios for implementing a decentralized microbiology diagnostic testing system.

**Results:**

There was an eight-month delay from the completion of the laboratory needs assessments to the initiation of sample collection due to the regional unavailability of appropriate supplies and equipment. When comparing study samples processed by study laboratory technicians and QC samples processed by other laboratory staff, there was 85.0% test result concordance for samples testing at the DHs and 90.0% concordance at the NRL. The cost for essential equipment and supplies for the three DHs was $245,871. The estimated costs for processing 600 samples ranged from $29,500 to $92,590.

**Conclusion:**

There are major gaps in equipment and supply availability needed to conduct basic microbiology assays at rural DHs. Despite these challenges, we demonstrated that it is feasible to establish microbiological testing capacity in Rwandan DHs. Building microbiological testing capacity is essential for improving clinical care, informing rational antibiotics use, and ultimately, contributing to the establishment of robust national antimicrobial stewardship programs in rural Rwanda and comparable settings.

## Introduction

Antimicrobial resistance (AMR) is a growing global health concern endangering progress in achieving infection control [1,2]. Although microbes naturally develop resistance, AMR is accelerated by the overuse or misuse of antimicrobial agents [3]. Treatment-resistant infections result in extended hospital stays and increased medical attention and require more complex and expensive treatment regimens, leading to an excess burden on the healthcare system and increased patient morbidity and mortality. These effects are magnified in resource-limited settings where trained staff, appropriate resources, and systems to guide informed patient care are scarce [4,5,6,7].

Antibiotic resistance profiling is critical for antimicrobial stewardship programs (ASPs). Data on antibiotic drug susceptibility of identified causal organisms enables targeted antimicrobial therapy [8]. To ensure the continuous and successful treatment and prevention of infectious diseases, the 2015 World Health Assembly adopted a global action plan on AMR that included improving awareness on AMR and strengthening evidence through surveillance and research as strategic priorities [9]. While data on pathogen profiles and AMR in sub-Saharan Africa (SSA) remain limited, the few existing studies conducted in urban or tertiary facilities show a trend of high pathogen diversity and widespread resistance [10,11,12,13,14,15].

In Rwanda, where this study was conducted, data on AMR are limited. A recent study from the University Teaching Hospital in Kigali indicated widespread prevalence of multi-drug resistant strains of *Escherischia coli, Klebsiella, Proteus*, and methicillin-resistant *Staphylococcus aureus* (MRSA) [16]. Additional studies in this area highlighted the need for more data on AMR patterns to inform rational infection control practices and to strengthen ASPs for improved patient outcomes, reduced development and spread of infections caused by multidrug-resistant organisms.

Infectious diseases remain a major public health concern globally, with low- and middle-income countries (LMICs) being disproportionally affected. In 2017, sepsis claimed 11 million lives worldwide, representing 19.7% of annual global deaths; 84.8% of these deaths occurred in LMICs [17]. Because AMR reduces the effectiveness of antimicrobials, patients who have compromised immunity are put at increased risk, including cancer patients and those undergoing surgical treatment. Infections can develop as acute or chronic complications of surgical or traumatic wounds and are often associated with diabetes, cancerous lesions, or vascular insufficiency [18]. In particular, cancer patients are more likely to develop multidrug-resistant infections due to prolonged hospitalizations and frequent need for antibiotics [19]. With a growing burden of non-communicable diseases (NCDs), the incidence of chronic wounds is likely to increase. Even with optimal treatment, infected wounds heal more slowly and are at risk of poorer healing processes and outcomes [20]. In high-income countries, culturing samples from wounds for pathogen identification and drug susceptibility testing are an integrated part of patient care and routine processes for guiding rational treatment plans. This differs from current practice in LMICs, where antibiotic use is largely not evidence-based due to a lack of trained staff and inadequate microbiology testing capacity [4].

To our knowledge, no previous study has assessed the feasibility of developing laboratory capacity for AMR research and surveillance in rural district hospitals (DHs) in SSA. To that end, we sought to assess the capacity of three DHs to collect and analyze wound swabs to inform clinical care. To complement this, we also describe our experience setting up microbiological diagnostic capacity at three DHs in rural Rwanda and costed equipment, supplies, and interventions implemented to conduct basic microbiological diagnostics.

## Methods

### Study sites

Partners In Health/Inshuti Mu Buzima (PIH/IMB), an international non-governmental organization (NGO) that began working in Rwanda in 2005, supports three rural DHs—Rwinkwavu District Hospital (RDH), Kirehe District Hospital (KDH), and Butaro District Hospital (BDH)—with a total catchment population of approximately 900,000 people. Each DH is located approximately two to three hours driving distance from the capital city, Kigali. This study was conducted at these three DHs, led by PIH/IMB in close collaboration with the Rwanda National Reference Laboratory (NRL).

In the Rwandan health system, DHs serve as an intermediary between health centers and provincial and tertiary referral hospitals. General practitioners (GPs), who have medical training equivalent to a U.S. medical school graduate, constitute the majority of physicians at DHs. Generally, DHs provide basic maternity, internal medicine, pediatric, emergency, out-patient care, and surgical care services. Although DHs are equipped to perform certain non-specialized diagnostic tests, the capacity to perform even basic microbiology testing, including capacity to collect and culture pathogens, is not present.

### Patient and public involvement

Patient and public involvement was not appropriate or possible during the design of the study, and due to the COVID-19 pandemic, it has also not been possible during the reporting and dissemination phase at the time of manuscript submission. Informed consent was obtained from all participating patients before performing study-related procedures.

### Wound assessment and surgical site infection studies

The AMR feasibility assessment was conducted through two sub-studies: (1) a wound assessment study (wound-AMR) and (2) a cesarean section surgical site infection (SSI) study (SSI-AMR). For the wound-AMR study, any patient over six months of age, presenting with a wound infection as determined by a hospital GP in the inpatient wards (internal medicine, surgery, maternity, pediatrics, or oncology), emergency department, or out-patient department (OPD) was eligible for enrollment. The study ran from September 1, 2019, to March 5, 2020, at BDH; October 1, 2019, to March 16, 2020, at KDH; and October 15, 2019, to March 16, 2020, at RDH. Data collection was originally scheduled to close on March 31, 2020, but the COVID-19 pandemic led to a premature termination of data collection. In parallel, an NIH-funded study (NIH R21TW011229) prospectively enrolled all women who underwent caesarean sections at KDH between September 23, 2019, and March 16, 2020. These women were assessed at postoperative day (POD) 11 (+/– 3 days) for an SSI, and those with an SSI were included in the SSI-AMR study. Both studies were approved by the Rwanda National Ethics Committee, and the Harvard Medical School Institutional Review Board also approved the SSI-AMR study. Both studies received technical and ethical approval from the PIH/IMB Research Committee and the three DHs prior to implementation. The manuscript was written in accordance with the Standards for Quality Improvement Reporting Excellence (SQUIRE) 2.0 guidelines.

We leveraged both studies to establish DH capacity for local AMR testing. The intention was to use the wound-AMR and SSI-AMR studies to establish long-term capacity for AMR testing at these facilities to support patient care, as well as hospital-level AMR surveillance. Below, we focus on the wound-AMR study, the goal of which was to establish capacity for sample collection and culturing at the DH, with pathogen and AMR profiling conducted at the NRL. The SSI-AMR samples were used to perform concordance assessments, and the use of these study results are described in detail below.

#### Baseline assessment

In collaboration with the NRL, using the World Health Organization’s guidelines for bacteriology in Biosafety Level 2 laboratories, we assessed the capacity to perform microbiology-related assays at the three DHs [21]. Based on findings from these assessments, a list of needed equipment and supplies was developed for each DH.

#### Standard operating procedures, sample collection, and culturing at DHs

Relevant bacteriology standard operating procedures (SOPs) on equipment (e.g., incubator, balance, etc.) maintenance and use, swab collection, sample processing, gram staining, and internal QC processes were adapted from existing NRL SOPs. The laboratory manager at each DH approved all SOPs prior to starting data collection.

De-identified data were collected using REDCap, a robust data collection tool that facilitates both online and offline data collection [22]. During specimen collection, study nurses obtained informed consent from study participants before collecting demographic information as well as specific details related to wound characteristics, prior antibiotic treatment, history of chronic diseases, and date and time of swab collection. Trained study nurses irrigated the wound using normal saline and, following irrigation, replaced their exam gloves with a pair of sterile gloves. A charcoal swab moistened with normal saline was then applied to a one centimeter area of clean viable tissue at the deepest accessible part of the wound. The swab was rotated for five seconds, using the Levine technique, to express discharge from the wound [23]. All swabs were immediately placed in tubes containing Amies transport media, and the wound was dressed by the hospital nursing staff per standard of care.

At each DH, two laboratory technicians were trained on culture media preparation, sample culturing, and sample handling processes, while bacterial identification and antibiotic susceptibility testing were conducted at the NRL in Kigali. To control for confounding factors that could result from differences in culture media preparation, agar plates were prepared at the NRL by the study laboratory technician and transported to study sites using triple packaging.

**Figure 1:**
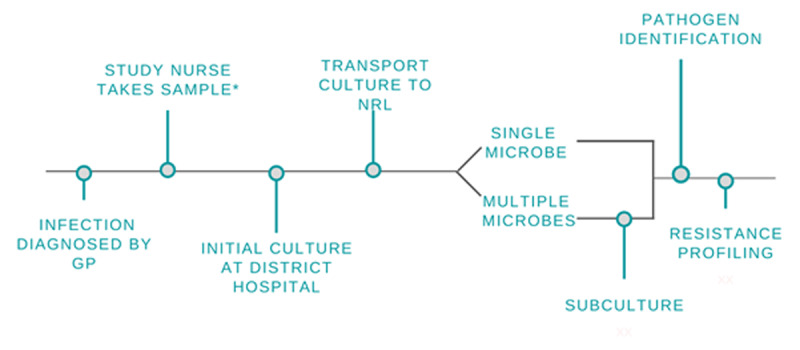
Overview of Study Processes. GP = General Practitioner. NRL = National Reference Laboratory. * The study process differed for the subgroup of women presenting with surgical-site infections enrolled in the parallel NIH-funded study where two swabs were collected: one swab was processed through this route and the other swab was sent directly to the NRL. This separate process is detailed in Figure 2.

The initial culturing of all samples was performed within 24 hours of sample collection. If the laboratory technician was unavailable in the 24 hours following swab collection, the swab was placed in the refrigerator for up to 24 hours to keep the samples viable. Initial cultures were incubated for 18–24 hours, examined by the laboratory technician for growth, and categorized as no growth, growth of a single microbe, or mixed growth before being sent to the NRL for further testing. If transport was delayed, cultures were stored in the refrigerator (+2 to +8 degrees Celsius) after incubation for up to 48 hours until transportation to the NRL was available (***Figure 1***).

#### Sample transport

Sample transportation to the NRL was scheduled twice a week, using standard transportation procedures with triple packaging and cold chain processes. To align with other transport logistics, the delivery of samples to the NRL was aligned with existing transportation schedules at each DH (Wednesdays and Fridays). Furthermore, to preserve the viability of collected samples, PIH/IMB cars facilitated the delivery of samples on Fridays. Thursdays were considered the last day to collect samples at each site to ensure all collected samples were transported to the NRL before expiration.

#### Bacterial isolation and identification

At the NRL, the study laboratory technician performed all bacterial isolation, identification, and antibiotic susceptibility testing (AST) in accordance with the NRL SOPs. The study laboratory technician examined initial cultures from the DHs for growth and categorized them as no growth, growth of a single microbe, or mixed growth. Cultures with mixed growth were sub-cultured for isolation of bacterial strains and further testing. Growth isolates were identified using colonial morphology, gram stain, and VITEK2 system testing. Because no universal recommended turnaround time (TAT) exists [24], we used the NRL recommendation of a maximum of seven days from the time of sample collection to generation of final results.

#### Antibiotic susceptibility testing

Drug susceptibility was done for each isolate using the VITEK2 antibiotic susceptibility testing system. Gram-positive bacteria were tested for susceptibility to penicillin, ampicillin, oxacillin, trimethoprim-sulfamethoxazole, tetracycline, ciprofloxacin, gentamicin, erythromycin, clindamycin, and vancomycin. Gram-negative bacteria were tested for susceptibility to ampicillin, piperacillin, amoxicillin-clavulanic acid, trimethoprim-sulfamethoxazole, tetracycline, ciprofloxacin, gentamicin, amikacin, ceftriaxone, cefepime, and imipenem.

#### Quality control

At each DH, two weeks of quality control (QC) data collection were conducted to assess the reliability of study results. At both the DHs and NRL, a second laboratory technician was involved in QC processes. At the DH, cultures from the study laboratory technician were compared with the ones from a hospital laboratory technician, who had the same training on culturing processes. For each initial culture, the DH laboratory technician assessed the growth of each culture as either no growth, growth of a single microbe, or mixed growth. Upon arrival at the NRL, the study laboratory technician documented the growth assessment of the same cultures. At the NRL, results from the study laboratory technician were compared with the ones from a senior laboratory technician in the bacteriology unit. To reduce the risk of bias, laboratory technicians involved in QC processes were not allowed to look at the other laboratory technicians’ work or communicate with each other about their results.

#### Agreement assessment

**Figure 2:**
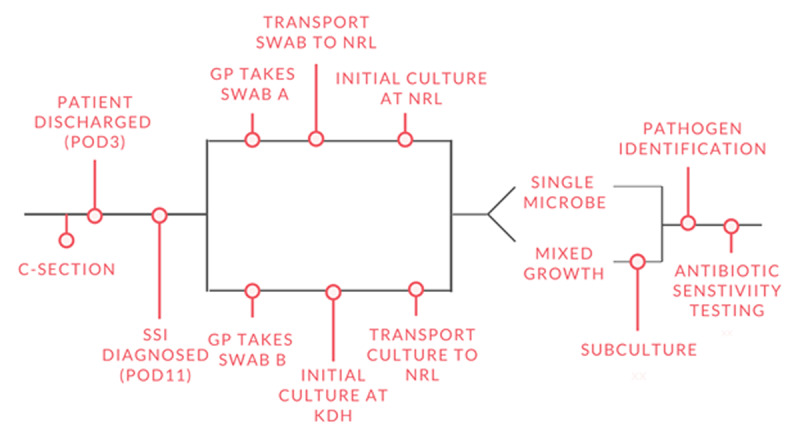
Processing of AMR-SSI samples from KDH. GP = General Practitioner. NRL = National Reference Laboratory.

Under the SSI-AMR study, all women undergoing caesarean sections at KDH who presented with an SSI at the study clinic on POD 11 (± 3 PODs) were swabbed twice. The first swab (sample A) was taken to the NRL within 24 hours to undergo initial culturing, pathogen identification, and AST. The second swab (sample B) followed the regular study process with initial culturing at the DH level and further sub-culturing and AST conducted at the NRL (***Figure 2***). The agreement between the results of sample A and B was taken as the second measure of microbiology diagnostic testing capacity at KDH. Full agreement was defined as the full concordance of all pathogen findings and antibiotic susceptibility. High agreement was defined as the full concordance of all pathogen findings but discrepancies in antibiotic susceptibility. Partial agreement was defined as the partial concordance of pathogen findings, while low agreement was defined as the concordance of gram-stain. No agreement was defined as no concordance of pathogen findings in the two samples.

#### Costing

Based on three different costing scenarios, we estimated the cost of pathogen identification and antibiotic susceptibility testing at the three DHs. For each scenario, estimates were done for the processing of 600 samples—the estimated annual case load based on the number of samples collected during the wound-AMR study. The first scenario considered all pathogen identification and sensitivity testing being conducted at the DH, the second scenario considered all testing being done at the NRL, and the third scenario considered a hybrid of the two scenarios, the approach used in this study. To estimate the costs, we compiled a list of the supplies (consumables) and equipment used to initiate the study with their respective costs to project the cost to collect and process 100 samples at one DH. Based on this list, the additional cost needed to process 600 samples was extrapolated.

## Results

### Baseline assessment

Initially, BDH had more equipment and supplies than RDH and KDH. RDH and KDH required purchasing of nearly all culturing equipment to establish basic hospital laboratory capacity needed for the study. Appendix I lists the per-sample supplies required for testing and our source for procurement. The most difficult item to procure was sheep’s blood used for blood agar plate preparations, which could only be procured from the U.S. After ordering it from the U.S., the sheep’s blood was contaminated during the initial shipment, and the subsequent shipment was delivered after 8–10 weeks, close to the product’s expiration date. At that time, a decision was made to use human blood from National Transfusion Center, per NRL protocol. Due to these delays in the requisition of essential supplies and equipment, it took eight months to begin data collection after the baseline assessment was completed.

### Sample Processing

**Figure 3:**
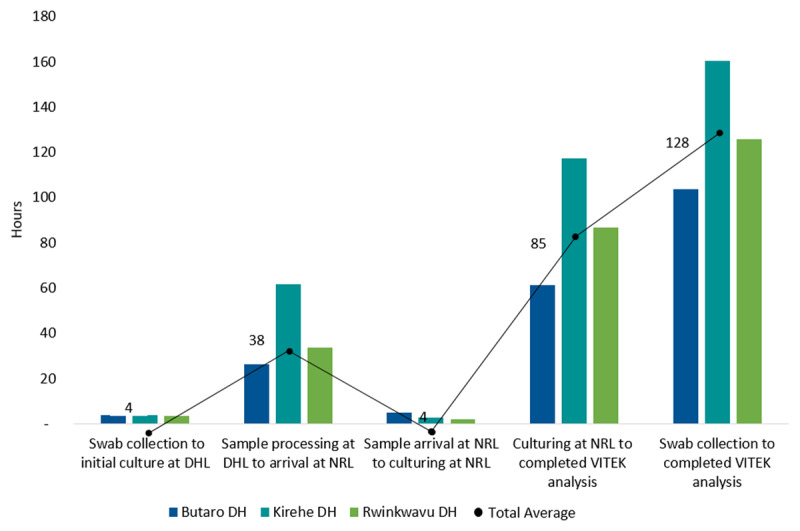
Turnaround Time, Disaggregated by Study Site and Sample Processing Steps. DHL = District Hospital Laboratory. NRL = National Reference Laboratory. DH = District Hospital.

Written informed consent was provided by 345 eligible patients who were enrolled at the 3 DHs for the wound-AMR study: 205 from BDH, 68 from KDH, and 72 from RDH. Among the 345 swab samples collected, 279 (80.9%) were initially cultured at the DHs. The remaining swabs were sent to the NRL directly due to the inability of the DH laboratory technician to process the sample in time (n = 34, 9.9%) or the unavailability of essential equipment (n = 32, 9.3%). The latter was the case at RDH, where the delay of one piece of equipment led the first 32 samples to be sent directly to the NRL for initial culturing. Overall, 343 samples (99.4%) underwent complete bacterial identification and antibiotic susceptibility testing at the NRL. The average turnaround time for sample processing, from swab collection to receipt of the final results from the NRL, was 128.4 hours, ranging from 104.0 hours at BDH to 160.0 hours at KDH (***Figure 3***). The most time-intensive step at all three hospitals was the period between culturing at the NRL and obtaining the VITEK system results. In total, 83.1% samples were fully processed within the recommended turnaround time of seven days.

### Quality control

Two weeks of QC were completed at BDH and RDH. The second week of QC data collection at KDH was not conducted because data collection was abruptly concluded due to the restriction measures put into place to curtail COVID-19 transmission. In total, 20 samples were collected for QC purposes, and the processes described above were used to assess the agreement between laboratory technicians at different levels (DH and NRL). At the DH level, concordance was 85.0%, and at the NRL, a 90.0% concordance was observed (***Table 1***).

**Table 1:** Concordance between Quality Control samples at the District Hospital (DH) and National Reference Laboratory (NRL).


QUALITY CONTROL AT DH LEVEL				

INITIAL CULTURE (A)	INITIAL CULTURE CONTROL (B)			

	*SINGLE MICROBE*	*MIXED GROWTH*	*NO GROWTH*	*TOTAL*

*Single microbe*	**12**	2	0	14

*Mixed growth*	0	**3**	0	3

*No growth*	1	0	**2**	3

**Total**	22	5	2	20

**Concordance**	(13+3+2)/20 = 17/20 **(85%)**			

**QUALITY CONTROL FOR SAMPLE A AT THE NRL**				

**INITIAL CULTURE (AA)**	**INITIAL CULTURE CONTROL (BA)**			

	***SINGLE MICROBE***	***MIXED GROWTH***	***NO GROWTH***	***TOTAL***

*Single microbe*	**14**	1	0	15

*Mixed growth*	0	**2**	0	2

*No growth*	1	0	**2**	3

**Total**	15	3	2	20

**Concordance**	(14+2+2)/20 = 18/20 **(90%)**			

**QUALITY CONTROL FOR SAMPLE B AT THE NRL**				

**INITIAL CULTURE (AB)**	**INITIAL CULTURE CONTROL (BB)**			

	***SINGLE MICROBE***	***MIXED GROWTH***	***NO GROWTH***	***TOTAL***

*Single microbe*	**14**	1	0	15

*Mixed growth*	0	**2**	0	2

*No growth*	1	0	**2**	3

**Total**	15	2	2	20

**Concordance**	(14+2+2)/20 = 19/20 **(90%)**			


Culture A: cultures prepared by the study laboratory technician at the DH. Culture B: cultures prepared by another laboratory technician at the DH. Culture AA: cultures prepared by the study laboratory technician at the DH and processed by the study laboratory technician at the NRL. Culture AB: cultures prepared by the study laboratory technician at the DH and processed by another laboratory technician at the NRL. Culture BA: cultures prepared by another laboratory technician at the DH and processed by the study laboratory technician at the NRL. Culture BB: cultures prepared by another laboratory technician at the DH and processed by another laboratory technician at the NRL.

### Concordance assessment

From the SSI-AMR study, 44 women with SSIs were swabbed with 2 samples (sample A and sample B) at KDH. Full agreement, defined as identical pathogen findings and antibiotic susceptibility results in sample A and B, was seen in 9.1% (n = 4) of the samples (***Table 2***). High agreement was seen in 47.7% (n = 21) of the samples; partial agreement was seen in 22.7% (n = 10) of the results, while 20.5% (n = 9) of the samples had low agreement.

**Table 2:** Concordance between AMR-SSI swabs processed through regular study processes and those taken directly to the National Reference Laboratory (NRL, N = 44).


LEVEL OF CONCORDANCE (N, %)				

FULL CONCORDANCE	HIGH CONCORDANCE	PARTIAL CONCORDANCE	LOW CONCORDANCE	NO CONCORDANCE

4 (9.1%)	21 (47.7%)	10 (22.7%)	6 (13.6%)	3 (6.8%)


Full concordance = full agreement of all pathogen findings and antibiotic susceptibility. High concordance = full agreement of all pathogen findings but discrepancies in antibiotic susceptibility. Partial concordance = full agreement of some pathogen findings. Low concordance = agreement of gram-stain. No concordance = no agreement of pathogen findings in the two samples.

### Costing

The total cost of the wound-AMR study was $245,871. Costs fell into three categories: personnel, equipment, and supplies. Approximately 17.0% ($40,891) of all costs were directed towards purchasing equipment to strengthen microbiology capacity at the three DHs. In terms of cost projections, if all processes for 600 samples are done at the DH level, including bacterial identification and antibiotic susceptibility testing, it would cost approximately $29,500 per site using manual sensitivity testing and approximately $92,590 using a VITEK2 system. The latter cost includes an estimated $47,727 for a one-time purchase of a VITEK2 machine for automated pathogen identification and sensitivity testing (***Table 3a***). In the second scenario, where processes are similar to our study processes, the cost would be approximately $34,452 (***Table 3b***). In the third scenario, where swabs would be taken straight to the NRL for all testing processes, costs would be slightly lower with an estimated cost of $33,770 per site (***Table 3c***).

**Table 3:** Costing estimates for processing 600 wound swabs from one district hospital (DH).


	*OPTION A: COST USING MANUAL TESTING AT DH*	*OPTION B: COST USING VITEK2 SYSTEM AT DH*

**A.**		

**Equipment**	13,695	13,695

**Laboratory supplies**	9,686	72,777*

**Reagents**	6,1193	6,119

**Total cost**	29,500	92,591

**B.**		

**Equipment and supplies at DH**	4,638	

**Reagents for NRL tests (pathogen identification and AST)**	29,814	

**Total cost**	34,452	

**C.**		

**Equipment and supplies at DH**	1,569	

**Reagents for NRL tests (pathogen identification and AST)**	32,201	

**Total cost**	33,770	


**a**: Scenario 1: Cost (USD) of having all pathogen identification and sensitivity testing done at DH. * This includes the cost of a VITEK machine to have tests done at DH. Costs not included: 1. Cost of one additional laboratory technician for sample processing. 2. Electricity, water, and other utility cost. **b**: Scenario 2: Cost (USD) of having initial cultures done at DH but samples sent to the NRL for pathogen identification and sensitivity testing. Costs not included: 1. Cost of one additional laboratory technician for sample processing at DH and NRL. 2. Cost of electricity, water, and other utility cost. 3. Cost for sample transportation. If culture media are prepared at the DHL, logistics for blood transportation from the national transfusion center to DHL should be taken into account. **c**: Scenario 3: Cost (USD) of having swabs sent from DH to the NRL for all pathogen identification and sensitivity testing. Costs not included: 1. Cost for having one additional laboratory technician for sample processing at the NRL. 2. Cost for electricity, water, and other utility cost. 3. Cost for sample transportation to NRL.

## Discussion

In this work, we demonstrate the feasibility of increasing AMR testing capacity at rural DHs and describe some of the challenges we encountered in Rwanda. In total, we processed 343 samples from 3 facilities, with an average time from sample collection to final results of 128.4 hours (~5.5 days). With respect to quality control, our team found a concordance between testing processes performed by study laboratory technicians and hospital laboratory personnel of above 80% at both levels (85% at DHs and 90% at NRL). This quality control assessment was made possible because the AMR-SSI study captured two swabs per patient, allowing for a comparison of the agreements of diagnostic assessments of samples taken from the same wound but processed using two different protocols.

We encountered several challenges, the most critical being related to procurement. Many items had to be procured internationally and were delivered to sites more than six months after the initial procurement request. This delayed the study overall and led to an ad-hoc restructuring of study processes for the first few weeks of data collection at RDH, from which all swabs were directly sent to the NRL.

One procurement challenge involved the procurement of sheep’s blood, the gold standard for laboratory analysis for bacterial culturing in high-income countries. With sheep’s blood not readily available in Rwanda and surrounding settings, we faced challenges, including contamination and short product shelf life. Ultimately, we decided to use human blood from the National Transfusion Center, which is the standard for the NRL. Human blood is frequently used in LMICs [25,26], even though limited modern microbiological research has been done to evaluate the reliability of results using human blood. Laboratory studies comparing human blood with sheep’s blood indicate that streptococcal species release cyclic AMP (cAMP), inhibiting hemolysis on human blood and potentially making detection of streptococcal species difficult [26,27,28]. Moving forward, more studies are needed to validate the use of human blood, which is currently the most feasible and sustainable alternative available in many low-income settings. In parallel, sites aiming to increase AMR testing capacity should consider developing mechanisms to locally source sheep’s blood, potentially through agreements between local sheep farms and hospital laboratories, as suggested numerous times to our team.

Another logistics challenge was determining the appropriate timing window for taking swabs, performing the initial cultures, and transporting them to the NRL to avoid deterioration of the quality of swabs or cultured plates. To ensure timely transport to the NRL, transport arrangements were made with DHs. Similarly, guidelines to ensure maintenance of cold-chain and biohazard safety standards were developed. Despite these arrangements, only 4.6% (n = 16) of swabs reached the NRL within a 24-hour time-window. Developing laboratory capacity in rural Rwanda entails challenges prevalent throughout similar low-resource settings. During the first week of data collection, the water system at BDH was not functioning appropriately, but the hospital availed distilled water necessary for the laboratory testing. Throughout the study period, all study sites experienced frequent power outages; however, all laboratory equipment were connected to devices such as refrigerator guards, power stabilizers, and uninterruptible power supply (UPS) systems that ensured continuous functioning even during periods of fluctuating electrical current or power outages.

As with any health system strengthening effort, the development of laboratory capacity had to be paired with a skills-building component. In the pre-implementation phase, we arranged trainings for the NRL study laboratory technician, six hospital laboratory technicians (two from each study site), three study nurses (one per site), and all head nurses at each site. Initially we trained only one laboratory technician per site, but during the implementation, we realized that this would limit our ability to perform all the processes within a reliable timeline and would make the study vulnerable to changing staff availability due to illness, conflicting hospital duties, or staff turnover. Thus, we worked with the DH leadership at each site to identify a second laboratory technician to be trained on the study processes. Due to the limited health workforce in Rwanda, as in many LMICs, some parts of the study implementation, such as QC, were delayed for several weeks due to scheduling conflicts for the laboratory technicians with other activities like national training workshops or the illnesses of study staff. Furthermore, staff turnover obliged us to have several refresher trainings throughout the duration of the study to appropriately onboard new study personnel on study processes.

Our costing assessment is consistent with other studies that have highlighted the considerable cost of carrying out microbiology diagnostics in low-resource settings given the limited health system infrastructure [29]. Although the cost of the first scenario, to have all testing performed at the DH level, is the highest ($92,591), this scenario would provide the most comprehensive microbiology testing capacity, including better point-of-care diagnostics to guide informed treatment plans. After the requisitioning of the VITEK machine, the cost for running 600 samples at the DH level would be approximately $44,863, which is close to the cost of the two other scenarios but with a potentially higher yield from a health system-strengthening point of view. Thus, the cost effectiveness of having comprehensive microbiology testing capacity at the DH level will require further investigation.

Another decentralization option not fully explored in this study is the development of microbiology capacity at the provincial level, where provincial hospitals act as intermediaries between DHs and NRL. This option would help to expand the testing capacity that currently exists only at the NRL to more rural areas, with each provincial hospital being connected to surrounding DHs. The use of provincial hospitals could potentially lessen the cost of scale-up by requiring less equipment and fewer trained staff, while still bringing microbiology diagnostics closer to the DH level. The creation of sentinel sites would expedite the identification of novel strains as well as delineating their sensitivity profiles, a critical asset for the rapid identification and control of outbreaks, as well as facilitating improved point-of-care diagnostic, patient care, and outcomes.

## Conclusion

Apart from BDH, which had some diagnostic equipment and supplies, there was largely no capacity to conduct microbiology assays at the DHs in our study, which are similarly, if not better, resourced than most comparable facilities in Rwanda. Strengthening microbiological capacity for pathogen identification and antibiotic susceptibility testing at DHs in rural Rwanda is feasible but entails multiple challenges. With regard to costing, setting up a microbiology service at a DH requires consideration of local and provincial infrastructural systems, such as reliable water and electricity supply in addition to facility level equipment, supply, and personnel costs. Investing in local AMR programs can provide a strong foundation for robust national AMR surveillance and antimicrobial stewardship programs and can help guide rational antibiotic prescription practices. Lessons learned in this study may inform and inspire the strengthening of microbiology capacity in comparable settings.

## Additional File

The additional file for this article can be found as follows:

10.5334/aogh.3416.s1 Supplement Data. All costs of AMR study.
